# Community awareness and knowledge of amyotrophic lateral sclerosis: a cross-sectional study in the Northern Border Region of Saudi Arabia

**DOI:** 10.7717/peerj.21710

**Published:** 2026-11-05

**Authors:** Raed AlRuwaili, Manair H. Alenzi, Mozon H. Alenzi, Abdulrahman Omar A. Alali, Samiah N. Aljohani, Randa F. Alenzi, Baraah Abu Alsel, Manal S. Fawzy

**Affiliations:** 1 Department of Internal Medicine, College of Medicine, Northern Border University, Arar, Saudi Arabia; 2 Faculty of Medicine, Northern Border University, Arar, Saudi Arabia; 3 Medical Sciences & Preparatory Year Department, North Private College of Nursing, Arar, Saudi Arabia; 4 Center for Health Research, Northern Border University, Arar, Saudi Arabia

**Keywords:** Amyotrophic lateral sclerosis, ALS awareness, Public knowledge, Community survey, Cross-sectional study, Northern Border Region, Saudi Arabia

## Abstract

**Background:**

Public awareness of amyotrophic lateral sclerosis (ALS) is limited worldwide, with potential implications for timely recognition, care seeking, and social support. Data from the Northern Border Region of Saudi Arabia are lacking. This study aims to assess community awareness and knowledge of ALS and identify predictors of good awareness among adults in the Northern Border Region of Saudi Arabia.

**Methods:**

A cross-sectional survey of 1,277 adults used a structured self-administered questionnaire covering sociodemographic characteristics, general awareness, knowledge, attitudes, personal experience, and information sources. Awareness was scored from 16 knowledge items and categorized as good (≥ 60%) or poor (<60%). Descriptive statistics, chi-square tests with Cramér’s V, and multivariable logistic regression were applied; model performance was assessed using the Hosmer–Lemeshow test and area under the receiver operating characteristic (ROC) curve (AUC).

**Results:**

Participants were mainly young to middle-aged, female, and university-educated, and most worked outside the health field. Although 45.2% had heard of ALS, 90.8% showed poor awareness, while only 9.2% showed good awareness. Uncertainty predominated regarding prognosis, risk groups, neuroanatomical involvement, non-motor symptoms, curability, and diagnostic difficulty. Age and occupation were associated with awareness in bivariate analyses but lost significance after adjustment. Never having heard of ALS markedly reduced the odds of good awareness (adjusted odds ratio (AOR) = 0.167, 95% confidence interval (CI) [0.097–0.288], *p* < 0.001), while using the internet and healthcare sources was associated with higher awareness. The model showed good calibration (Hosmer–Lemeshow *p* = 0.891) and discrimination (AUC = 0.820).

**Conclusions:**

Community awareness and knowledge of ALS in this region are poor, underscoring the need for targeted digital and healthcare-based educational initiatives to improve ALS literacy.

## Introduction

Amyotrophic lateral sclerosis (ALS) is a progressive, fatal neurodegenerative disorder characterized by degeneration of upper and lower motor neurons, leading to progressive muscle weakness, respiratory failure, and death, typically within 3–5 years of symptom onset (Brotman et al., 2024). Although relatively rare, ALS imposes a disproportionate clinical and societal burden due to its rapid course, lack of curative therapy, and complex care needs that require coordinated multidisciplinary services (Artioli et al., 2025). The global incidence is estimated at approximately 2–3 per 100,000 person-years, with increasing case numbers as populations age (Vasta et al., 2022; Wolfson et al., 2023).

Timely recognition of ALS and early referral to specialist care can optimize symptom management, extend survival, and support advanced care planning (Reynolds & Chhetri, 2024). While the formal diagnosis of ALS depends on specialist assessment and investigations, community awareness may still influence how quickly individuals seek medical attention for concerning neurological symptoms and how societies prioritise support for affected patients and caregivers, by analogy with other neurological conditions such as stroke and epilepsy (Alzayer et al., 2023; Aytaç et al., 2014; Mangal et al., 2024). Previous studies have shown that public awareness of motor neuron disease is often poor, with frequent confusion between ALS and other neurological disorders such as multiple sclerosis and Parkinson’s disease, misconceptions about prognosis and etiology, and limited knowledge of core symptoms (Celius et al., 2021; Crooks et al., 2025; Davies & Turner, 2010). These gaps may contribute to delays in help-seeking and weaker societal and economic support for patients, caregivers, and ALS services, although robust data directly linking ALS awareness to earlier diagnosis and advocacy remain limited (Song, 2014). However, empirical evidence on whether greater community awareness and knowledge of ALS translates into improved patient care or enhanced social and economic support for caregivers remains scarce, highlighting the need for baseline data to inform future interventions. In Saudi Arabia, emerging data indicate substantial challenges in ALS care provision, including inconsistent access to recommended multidisciplinary measures; however, little is known about community-level awareness of ALS (Abuzinadah et al., 2020; Alshoshan et al., 2026). Improving community awareness is therefore only one component of a broader strategy; it must be complemented by healthcare pathways that allow rapid access to neurological assessment and multidisciplinary ALS care when symptoms arise (Song, 2014). Understanding public knowledge, attitudes, and information sources is essential to inform targeted educational strategies and national planning for neurological services (Almarwani et al., 2025).

Therefore, this study aimed to assess community awareness and knowledge of ALS and to identify factors associated with good awareness among adults in the Northern Border Region of Saudi Arabia.

## Materials & Methods

### Study design and setting

This cross-sectional, community-based study was conducted in the Northern Border Region of Saudi Arabia, an area located in the north of the Kingdom with a mixed urban and semi-urban population (Alenzi et al., 2023). According to recent national statistics (based on the 2022 Census), the Northern Border Region has an estimated population of approximately 202,712 residents (City Population, 2022). Data were collected between 19 and 29 November 2025 using a structured questionnaire administered to adults residing in the region.

### Study population and eligibility criteria

The target population comprised adults aged 18 years or older living in the Northern Border Region. Individuals were eligible if they were residents of the region, could read and understand Arabic (the region’s mother tongue), and consented to participate. Healthcare professionals, health sciences students, non-health students, employed individuals, unemployed adults, and retirees were all included to capture community-level awareness.

### Sample size calculation

The minimum required sample size for this cross-sectional survey was estimated using the standard formula for a single population proportion: *n* = *Z* ^2^*p(1*−*p)/d* ^2^, assuming a two-sided 95% confidence level (*Z* = 1.96), an anticipated awareness proportion *p* = 0.50 (to maximize sample size in the absence of prior regional data), and a margin of error *d* = 0.03 (Egbuchulem, 2023). This yielded a minimum sample size of approximately *n* = 1,067. To account for potential non-response and incomplete questionnaires, we inflated this figure by about 20%, resulting in a target sample of about 1,280 participants. Ultimately, 1,277 fully completed questionnaires were available for analysis and included in the final dataset.

### Sampling and recruitment

A non-probability, convenience sampling strategy was employed, recruiting adults from public places (*e.g.*, shopping centers, workplaces, educational institutions) and *via* electronic distribution of the questionnaire through social media platforms commonly used in Saudi Arabia (Al-Hamdan et al., 2021). Trained data collectors introduced the study, confirmed eligibility, obtained written informed consent, and provided instructions for completing the questionnaire. For the online version, an introductory page detailed the study purpose, inclusion criteria, and the voluntary nature of participation. Before proceeding, written consent was obtained from all study participants.

### Questionnaire development and content

The study used a structured, self-administered questionnaire designed to assess community awareness and knowledge of ALS. The instrument was developed based on the literature on public awareness of motor neuron disease, ALS, and other neurological disorders, and adapted from previously published tools with appropriate contextual modifications (Davies & Turner, 2010). The final questionnaire consisted of five sections: (1) sociodemographic characteristics (age, gender, education level, occupation, and region of residence). (2) General awareness of ALS: prior hearing of ALS, confusion with multiple sclerosis or Parkinson’s disease, and awareness/participation in the ALS Ice Bucket Challenge (*i.e.,* a viral social media campaign launched in 2014 in which participants poured ice water over their heads to promote ALS awareness and encourage charitable donations). (3) Knowledge of ALS characteristics and symptoms: items on expected survival, sex and age at highest risk, primary affected system (brain/spinal cord *vs.* muscles and others), perceived etiology (genetic, lifestyle, infectious, unknown), and core symptoms (muscle weakness, dysarthria, dysphagia, respiratory difficulty, sensory loss, memory loss), as well as knowledge of curability and diagnostic difficulty. (4) Attitudes and perceptions: perceived seriousness of ALS, perceived stigma in Saudi culture, perceived adequacy of information provided by official campaigns/media, and interest in learning more about ALS. (5) Experience and information sources: personal or vicarious experience with ALS (knowing a patient, caring for a patient, knowing a famous person with ALS) and main sources of health- and ALS-related information (internet, social media, TV/radio, healthcare professionals, family/friends, other).

Where relevant, questions were answered on categorical or Likert-type scales. The questionnaire was initially developed in English and then translated into Arabic, with back-translation to ensure semantic equivalence. A pilot test was performed on a small convenience sample from the same region to assess clarity, cultural appropriateness, and completion time; minor wording adjustments were made accordingly.

### Awareness scoring

Awareness was quantified using 16 knowledge items covering ALS identity, prognosis, risk groups, affected system, symptoms, etiology, curability, and diagnostic difficulty. Each correct answer was scored 1, and each incorrect or “I don’t know” answer was scored 0, yielding a total score ranging from 0 to 16, consistent with scoring schemes used in other public awareness surveys. In line with prior awareness and knowledge, attitude and practice (KAP) studies that apply ’Bloom’s cutoff to dichotomize knowledge levels, overall awareness was categorized as good when participants achieved a score ≥60% (≥10 correct answers) and poor when the score was <60% (<10 correct answers)(Esmaeel et al., 2026a; Esmaeel et al., 2026b; Feleke, Wale & Yirsaw, 2021).

### Ethical considerations

The study was conducted in accordance with the principles of the Declaration of Helsinki and approved by the “Research Ethics Committee (REC) of North Private College of Nursing (approval no. NCN-10112025-90)”. Participation was voluntary, and completion of the questionnaire implied informed consent. All data were collected anonymously and treated confidentially; no personal identifiers were recorded.

### Data management and statistical analyses

The collected raw data are provided in Supplementary Data File S1. Data were managed and analyzed using IBM SPSS Statistics version 20.0. Descriptive statistics were used to summarize the characteristics of the 1,277 participants, including sociodemographic variables, awareness, knowledge items, attitudes, experiences, and sources of information. Categorical variables were presented as frequencies and percentages. Overall awareness was categorized as good or poor based on participants’ responses, with scores ≥60% considered good and <60% considered poor; specifically, good awareness was defined as correctly answering at least 10 out of 16 knowledge-related questions.

The chi-square test was used to examine associations between overall awareness and demographic characteristics (age group, gender, education level, occupation), with *p*-values <0.05 considered statistically significant. Cramér’s V was calculated to quantify effect size for significant associations. Variables with *p* < 0.20 in bivariate analyses or judged clinically relevant were entered into a multivariable logistic regression model to explore factors independently associated with good awareness. The dependent variable was overall awareness (good *vs.* poor), and independent variables included age group, gender, education level, occupation, prior hearing of ALS, and key information sources (internet, TV, healthcare professionals, family, and media/social platforms). Model results were reported as adjusted odds ratios (AORs) with 95% confidence intervals (CIs) and corresponding *p*-values; *p*-values <0.05 were considered statistically significant.

Model fit was assessed using the Hosmer–Lemeshow goodness-of-fit test, and the area under the receiver operating characteristic (ROC) curve (AUC) was reported descriptively to summarize the model’s ability to distinguish between participants with good and poor awareness. Cook’s Distance was examined to detect potentially influential observations; values approaching or exceeding 1 were considered potentially influential.

## Results

### Sociodemographic characteristics

A total of 1,440 questionnaire responses were received. For the present analysis, only participants who (i) provided consent, (ii) reported residing in the Northern Border Region, and (iii) had sufficiently complete data on the main awareness items were included to yield a final analytic sample of 1,277 adults. Females comprising the majority of the sample (66.2%, *n* = 845), and males represented 33.8% (*n* = 432).

Participants were predominantly younger and middle-aged, with 37.7% aged 18-30 years and 31.6% aged 41-50 years. Those aged 31–40 years accounted for 17.9%, and 12.9% were aged 50 years or older (Fig. S1).

Most participants held a bachelor’s degree (65.3%), and those with less than a high school education accounted for 3.6%. Regarding occupation, 42.4% worked outside the health field, while 14.8% were health sciences students, and 6.2% were retired (Table 1).

**Table 1:** Education level and occupation of participants (*N* = 1,277). Data are presented as frequencies (n) and percentages (%).

**Variable**		**Frequency**	**Percent (%)**
Education level	Less than high school	46	3.6
	High school graduate	179	14.0
	Diploma	139	10.9
	Bachelor’s degree	834	65.3
	Postgraduate degree	79	6.2
Occupation	Health Sciences student	189	14.8
	Non-Health Sciences student	91	7.1
	Work (in the health field)	71	5.6
	Work (out of the health field)	541	42.4
	Not work	306	24.0
	Retired	79	6.2

**Notes.**

Data are presented as frequencies (*n*) and percentages (%).

**Figure 1:**
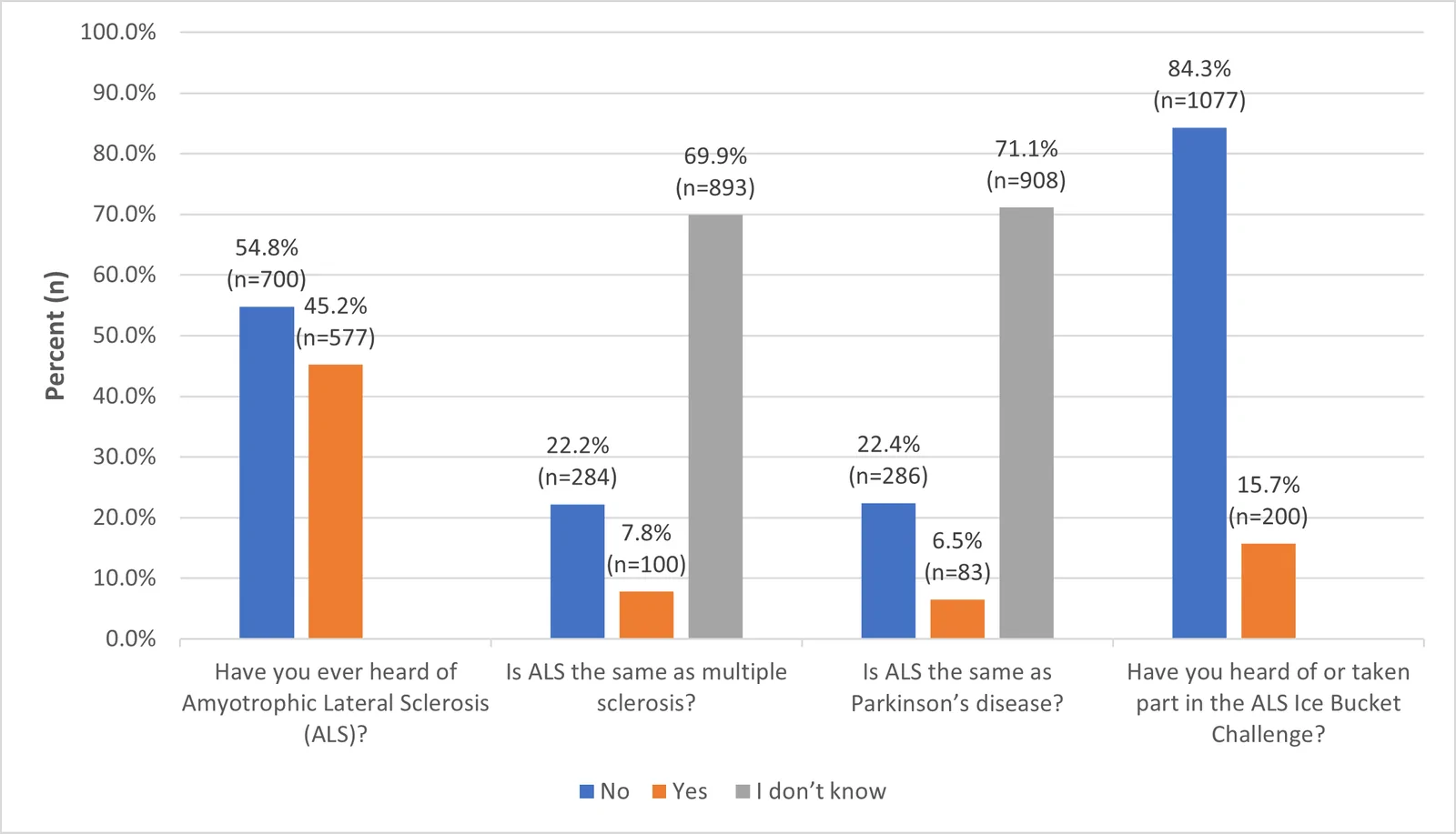
Awareness of ALS among participants (*N* = 1,277).

### General awareness of ALS

Less than half of the participants had ever heard of ALS (45.2%), whereas 54.8% reported no prior awareness. Knowledge of ALS in relation to other neurological conditions was limited: 69.9% were unsure whether ALS is the same as multiple sclerosis, and 71.1% were unsure about Parkinson’s disease, with only 22.2% and 22.4%, respectively, correctly responding that ALS is distinct. Awareness of the ALS Ice Bucket Challenge was low: 15.7% reported participation, and 84.3% indicated they had not participated (Fig. 1).

### Knowledge of ALS characteristics

Overall knowledge of ALS was generally limited. Most participants did not know the expected survival of a person with ALS (65.5%), while 9.1% estimated 5 years, 9.6% estimated 5–10 years, and 15.7% believed life expectancy is normal. For sex distribution, 63.4% did not know which sex is most affected, whereas 22.5% correctly identified males and 14.1% selected females.

Regarding age-related risk, 54.3% were unsure which age group is at the highest risk; 26.9% correctly identified the 40–70 years age group, 16.4% chose <40 years, and 2.4% chose>70 years. When asked which system ALS primarily affects, 41.7% responded “I don’t know”, 28.9% correctly selected the brain and spinal cord, 27.6% selected muscles, and only 1.1% and 0.8% chose the heart or skin, respectively (Table 2).

**Table 2:** Knowledge of ALS characteristics among participants.

**Item**	**Category**	**Frequency**	**Percent (%)**
How long is a person with ALS likely to live?	5 years	116	9.1%
	5–10 years	123	9.6%
	Normal life expectancy	201	15.7%
	I don’t know	837	65.5%
Which gender is most affected by ALS?	Male	287	22.5%
	Female	180	14.1%
	I don’t know	810	63.4%
Which age group has the highest risk of developing ALS?	<40	209	16.4%
	40–70	343	26.9%
	>70	31	2.4%
	I don’t know	694	54.3%
ALS primarily affects which of the following?	Muscles	352	27.6
	Brain and spinal cord	369	28.9
	Heart	14	1.1
	Skin	10	.8
	I don’t know	532	41.7

**Notes.**

Data are presented as frequencies (*n*) and percentages (%).

### Perceived etiology of ALS

Perceptions of ALS etiology were heterogeneous. Genetic causes were selected by 35.8% of participants, and lifestyle-related factors such as obesity, smoking, and drug use by 28.7%. Infectious causes were selected by 7.1%, 24.7% acknowledged that the cause is not known, and 40.3% responded “I don’t know” (Fig. S2).

### Knowledge of ALS symptoms, cure, and diagnosis

Understanding of ALS symptoms was limited. Muscle weakness was the most commonly recognized symptom (55.2%), but 42.1% remained unsure. Dysarthria and dysphagia were recognized by 38.9% and difficulty breathing by 31.3%, with the majority selecting “I don’t know” for these items. Only 30.2% correctly identified that ALS does not cause loss of sensation, and 16.0% that it does not cause memory loss (Fig. 2).

Uncertainty also extended to treatment and diagnosis. Most participants did not know whether a cure exists (67.6%); 19.1% correctly indicated that no cure is currently available, while 13.3% believed that a cure exists. Similarly, 62.6% were unsure about the difficulty of diagnosing ALS, whereas 22.7% found it difficult and 14.7% found it not difficult (Fig. S3).

**Figure 2:**
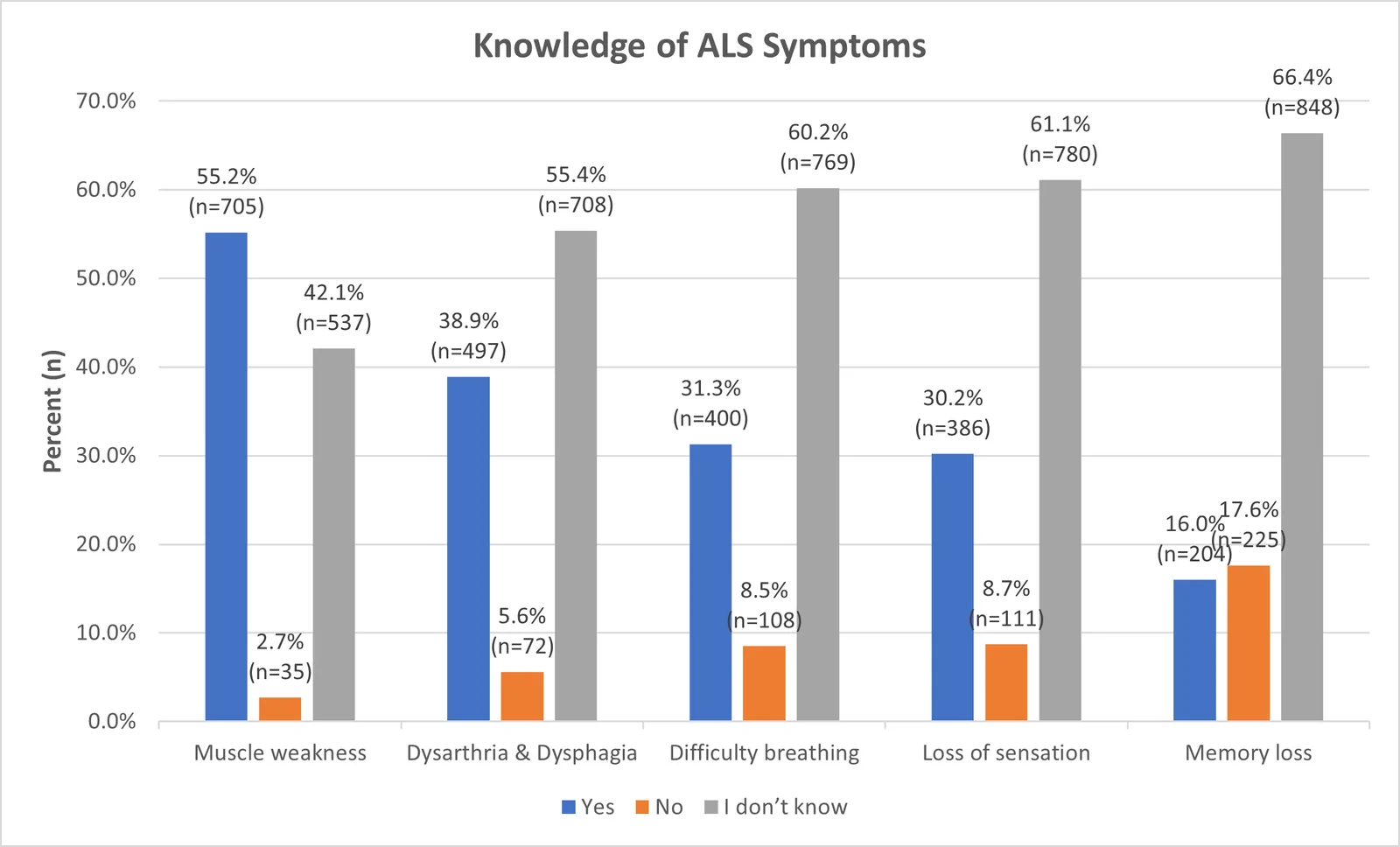
Knowledge of ALS symptoms among participants.

### Attitudes toward ALS and perceived stigma

Most respondents regarded ALS as a serious disease. Overall, 38.1% rated it as very serious and 14.6% as moderately serious, while 42.0% were uncertain; only 4.5% considered ALS slightly serious and 0.9% not serious (Fig. S4).

Perceptions of stigma and information availability were mixed. More than half of participants were unsure whether ALS carries stigma in Saudi culture (55.8%), 26.0% believed there is no stigma, and 18.2% perceived that stigma exists. Regarding information availability, 39.5% felt that official health campaigns or media do not provide enough information, 20.1% believed information is sufficient, and 40.3% were unsure. Interest in learning more about ALS was high: 62.9% expressed interest, 30.3% were unsure, and only 6.8% were not interested (Fig. 3).

**Figure 3:**
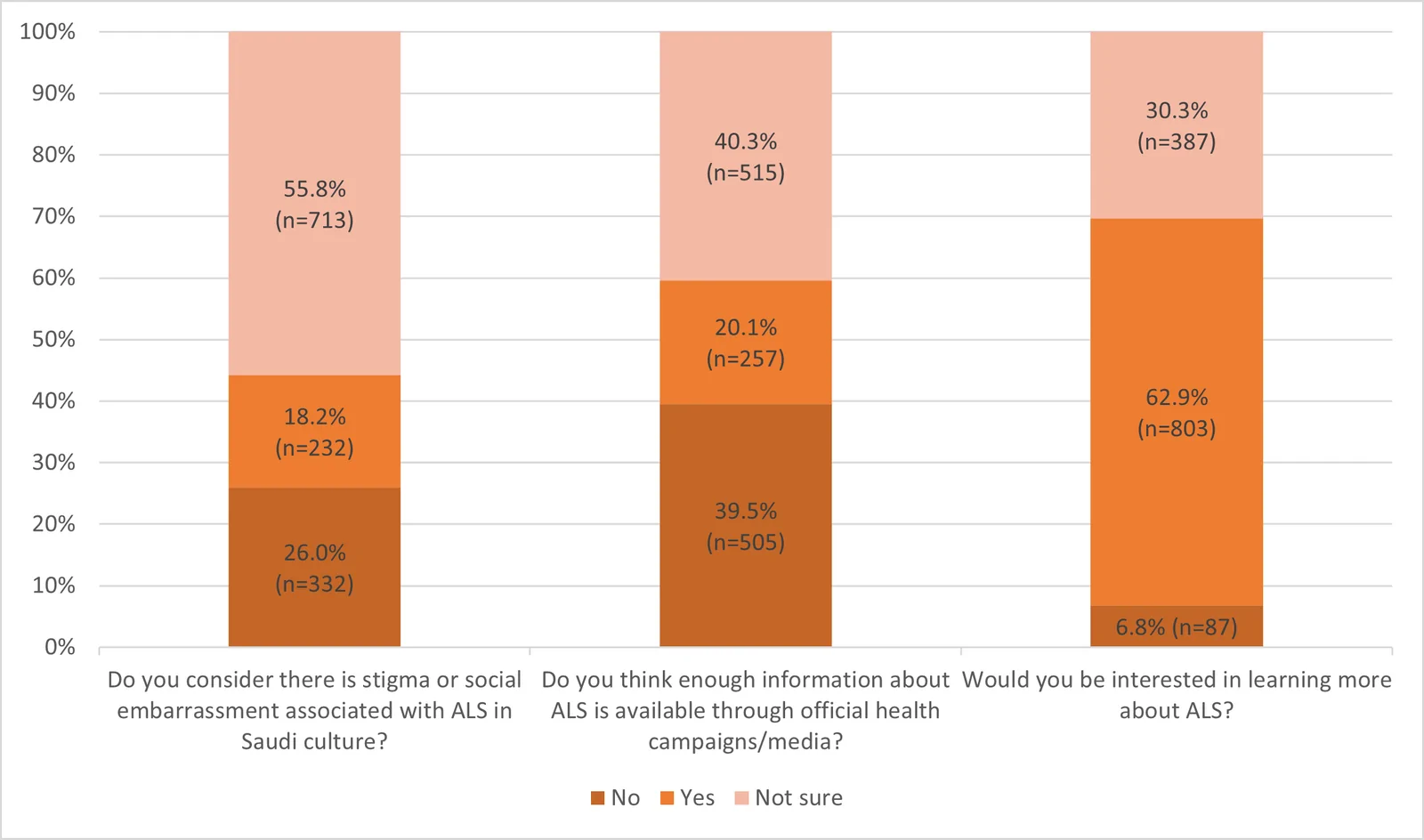
Attitudes of ALS stigma, information availability, and interest in learning more.

### Personal and vicarious experience with ALS

Most participants (87.5%) reported not knowing any famous person in Saudi Arabia diagnosed with ALS, whereas 12.5% indicated that they did. Personal contact with ALS was also limited: 16.0% knew someone diagnosed with ALS, and 8.7% had ever cared for or supported an affected person (Fig. S5).

### Sources of information about ALS

The internet was the most frequently reported source of information (44.2%), followed by social media (36.6%) and family or friends (22.3%). Healthcare professionals were identified as a source by 15.3% of participants, and TV/radio by 7.5% (Fig. S6).

### Overall level of awareness

Overall, 1160 (90.8%) of participants were classified as having poor awareness of ALS, while only 117 (9.2%) achieved good awareness according to the predefined scoring criteria.

### Bivariate associations between demographics and awareness

Age group and occupation were significantly associated with overall awareness level. Participants aged 18–30 years had a higher proportion of good awareness compared with older groups (Chi-square = 23.692, *p* < 0.001; Cramér’s V = 0.136). Health sciences students showed the highest awareness among occupational categories (Chi-square = 37.373, *p* < 0.001; Cramér’s V = 0.171), whereas gender and education level were not significantly associated with awareness (*p* > 0.05) (Table 3).

**Table 3:** Association between demographic variables and overall awareness level.

**Variables**	**Category**	**Overall Awareness Level**		**Chi-Square** **(*P*. value)**	**Cramér’s V** **(*P*. value)**
		**Poor**	**Good**		
Age Group	18–30	413 (35.6%)	68 (58.1%)	23.692 (<0.001)	0.136 (<0.001)
	31–40	215 (18.5%)	13 (11.1%)		
	41–50	380 (32.8%)	23 (19.7%)		
	>50	152 (13.1%)	13 (11.1%)		
Gender	Male	400 (34.5%)	32 (27.4%)	2.415 (0.120)	0.043 (0.120)
	Female	760 (65.5%)	85 (72.6%)		
Education level	Less than high school	44 (3.8%)	2 (1.7%)	1.960 (0.743)	0.039 (0.743)
	High school graduate	161 (13.9%)	18 (15.4%)		
	Diploma	126 (10.9%)	13 (11.1%)		
	Bachelor’s degree	759 (65.4%)	75 (64.1%)		
	Postgraduate degree	70 (6.0%)	9 (7.7%)		
Occupation	Health Sciences student	154 (13.3%)	35 (29.9%)	37.373 (<0.001)	.171 (<0.001)
	Non-Health Sciences student	78 (6.7%)	13 (11.1%)		
	Work (in the health field)	60 (5.2%)	11 (9.4%)		
	Work (out of the health field)	513 (44.2%)	28 (23.9%)		
	Not work	282 (24.3%)	24 (20.5%)		
	Retired	73 (6.3%)	6 (5.1%)		

**Notes.**

Significance was set at *p* < 0.05.

When occupation was categorized as health *versus* non-health, good awareness was more common in the health group (17.7%) than in the non-health group (7.0%). Poor awareness was more prevalent among those without a health background (93.0% *vs.* 82.3%), with a small-to-moderate effect size (Chi-square = 28.543, *p* < 0.001; Cramér’s V = 0.150, *p* < 0.001).

### Multivariable factors associated with awareness and model diagnostics

In the multivariable logistic regression model, age, gender, education level, and occupation were not significantly associated with overall awareness; adjusted odds ratios were close to 1 with *p*-values > 0.05. Although age and occupation showed significant associations in bivariate analyses, these effects disappeared after adjustment, suggesting confounding by more proximal factors such as prior hearing of ALS and access to health-related information (Table 4).

**Table 4:** Logistic regression analysis of factors influencing overall awareness.

**Variable**	**Comparison**	***p*-value**	**Adjusted odds ratio (AOR)**	**95% Confidence interval**
Age	31–40 *vs.* 18–30	0.865	0.924	0.370–2.305
	41–50 *vs.* 18–30	0.372	0.642	0.243–1.696
	>50 *vs.* 18–30	0.784	0.891	0.390–2.035
Sex	Female *vs.* Male	0.414	0.807	0.482–1.350
Education	Less than high school *vs.* Postgraduate	0.134	0.268	0.048–1.497
	High school graduate *vs.* Postgraduate	0.282	0.579	0.214–1.566
	Diploma *vs.* Postgraduate	0.680	0.805	0.287–2.256
	Bachelor’s *vs.* Postgraduate	0.335	0.661	0.285–1.533
Occupation	Non-Health Sciences student *vs.* Health Sciences student	0.371	1.803	0.495–6.562
	Work (in the health field) *vs.* Health Sciences student	0.473	1.618	0.435–6.015
	Work (out of the health field) *vs.* Health Sciences student	0.477	1.629	0.424–6.254
	Not a work *vs.* Health Sciences student	0.637	0.768	0.258–2.293
	Retired *vs.* Health Sciences student	0.808	1.160	0.351–3.833
Previous hearing about ALS	None heard *vs.* Heard	0.000	0.167	0.097–0.288
Source of information	No use Internet vs Use Internet	0.000	0.384	0.244–0.602
	No use TV vs Use TV	0.481	0.789	0.408–1.525
	No use Healthcare sources vs Use	0.005	0.498	0.306–0.808
	No use Family sources vs Use	0.292	0.773	0.478–1.249
	No use Media sources vs Use	0.229	0.768	0.499–1.181

Prior exposure to ALS and the use of specific information sources emerged as strong independent correlates of good awareness. Participants who had never heard of ALS had markedly lower odds of good awareness than those who had (AOR = 0.167, 95% CI [0.097–0.288], *p* < 0.001). Using the internet (AOR = 0.384, 95% CI [0.244–0.602], *p* < 0.001) and healthcare sources (AOR = 0.498, 95% CI [0.306–0.808], *p* = 0.005) as main channels for health and ALS-related information were significantly associated with higher awareness, whereas TV, family, and other media were not significantly associated with awareness in the adjusted model (Table 4).

Model fit was acceptable according to the Hosmer–Lemeshow test (Chi-square = 3.61, *df* = 8, *p* = 0.891), indicating no evidence of lack of fit. The ROC analysis yielded an AUC of 0.820 (SE = 0.018, *p* < 0.001; 95% CI [0.79–0.86]), suggesting that the model distinguished reasonably well between participants with good and poor awareness (Fig. 4). The ROC curve lay well above the diagonal reference line, indicating a favorable trade-off between sensitivity and specificity across thresholds. Cook’s Distance values indicated no problematic influential observations, with a mean of 0.132 (SD = 0.101) and a range from 0.002 to 0.564, all below the conventional cutoff of 1.

**Figure 4:**
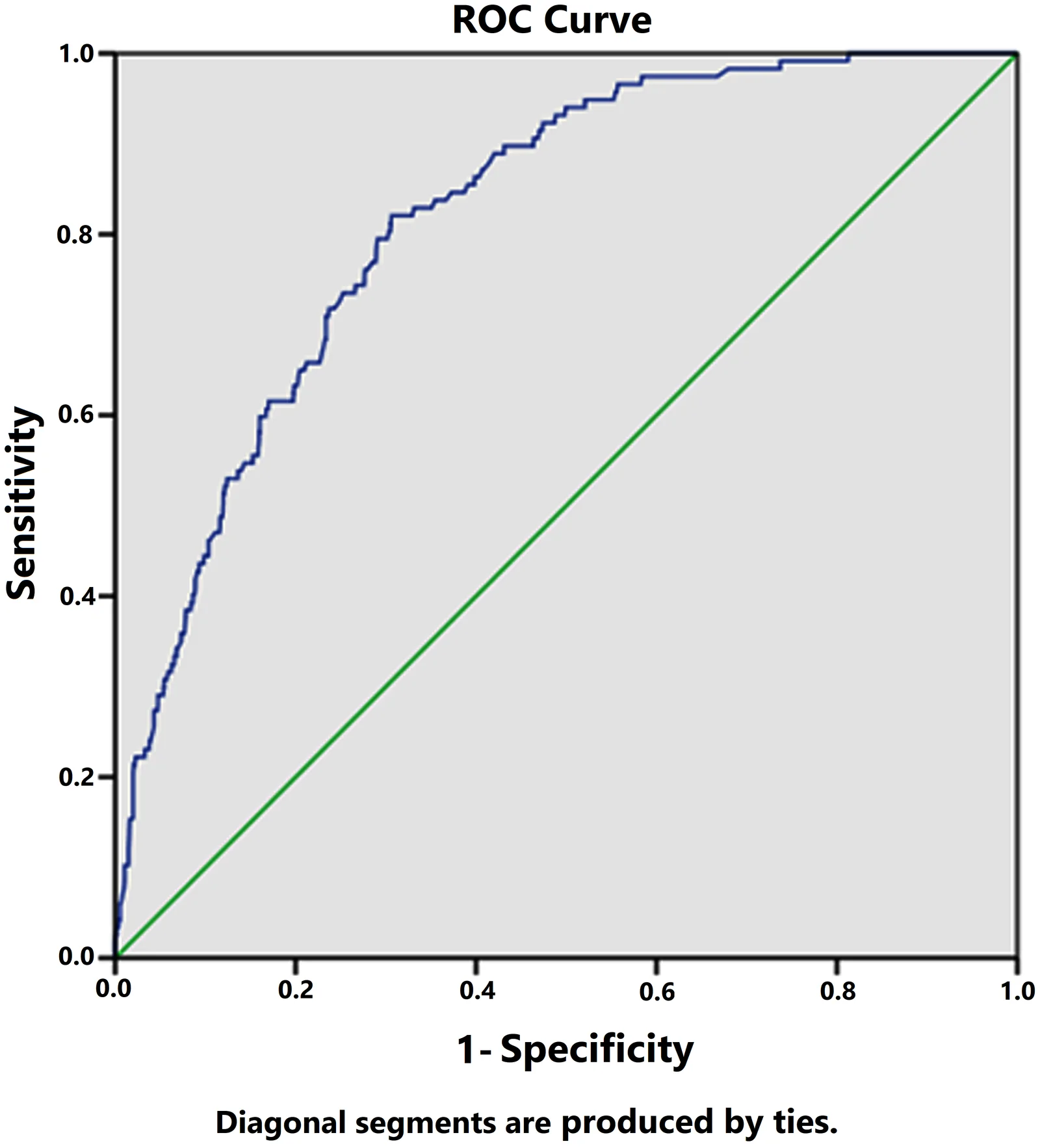
ROC curve for the multivariable model of ALS awareness.

## Discussion

ALS awareness levels in the current community were strikingly low, despite the disease’s severity and the region’s relatively young, educated population. Fewer than half of the participants had ever heard of ALS. Over 90% fell into the poor-awareness category, mirroring earlier work from the UK and Pakistan showing that motor neuron disease (MND) is poorly recognized by the general public and often confused with other neurological conditions (Chau et al., 2025; Davies & Turner, 2010). These findings suggest that ALS remains largely invisible as a public health issue, even though it imposes a disproportionate clinical and socioeconomic burden and requires complex multidisciplinary care (Abuzinadah et al., 2020).

The pattern of knowledge gaps in this study, uncertainty about prognosis, age and sex at risk, the primary neuroanatomical substrate, and non-motor manifestations, aligns with previous surveys indicating that laypeople rarely appreciate ALS as a progressive, incurable motor neuron disorder with profound functional, psychosocial, and palliative implications (Abuzinadah et al., 2020). The high frequency of “I don’t know” responses regarding etiology and cure may reflect the genuine scientific uncertainty about ALS pathogenesis and the absence of disease-modifying therapies. Still, it also highlights missed opportunities for conveying clear messages about established risk factors, symptomatic treatments, and the role of multidisciplinary clinics in optimizing quality of life (Harris et al., 2017; Levison, Blicher & Andersen, 2024; Turner & Talbot, 2013; NICE guideline No. 42, 2019).

Importantly, our data show that demographic variables alone are poor discriminators of awareness, once prior exposure and information sources are taken into account. Age and occupation were associated with awareness in chi-square analyses, with higher awareness among younger adults and health sciences students, consistent with other neurological awareness studies in Saudi Arabia; however, these associations disappeared in the multivariable model, suggesting confounding by more proximal factors such as prior hearing of ALS and access to health-related information. This aligns with work on multiple sclerosis and other neurological conditions, showing that “having heard of the disease” and prior contact with affected individuals are more powerful predictors of knowledge than sociodemographic characteristics per se (Al-Hamdan et al., 2021; Dahlawi et al., 2022; Elmosaad et al., 2025).

By contrast, prior exposure to ALS and the use of the internet and healthcare sources emerged as strong independent predictors of good awareness in our model. Participants who had never heard of ALS had markedly lower odds of adequate knowledge. At the same time, those using online and healthcare-professional channels were significantly more likely to be knowledgeable, echoing studies that found social media, web-based content, and professional counseling were the dominant sources of information about MND and ALS (Abuzinadah et al., 2020; Al-Hamdan et al., 2021; Dahlawi et al., 2022). These findings have direct implications for intervention design: digital platforms and clinician-led education are likely to be the most efficient levers for improving ALS literacy in this setting, particularly if campaigns are tailored to regional languages and health-literacy levels (Dong et al., 2023; Fitzpatrick, 2023; Mukhtar et al., 2025; Sutrisman et al., 2025).

The high proportion of participants perceiving ALS as a serious disease, despite limited factual knowledge, indicates a partially formed risk perception that targeted campaigns could harness. Analogous work in multiple sclerosis, epilepsy, and other neurological conditions in Saudi Arabia has shown that low knowledge coexists with fear and stigma, and that structured awareness initiatives can improve understanding and reduce negative attitudes (Abuzinadah et al., 2020; Al-Hamdan et al., 2021; Dahlawi et al., 2022; Haneef et al., 2014; Khan, 2015). In our study, more than half of the respondents were unsure about ALS-related stigma and considered current information from official campaigns insufficient, yet most expressed interest in learning more, underscoring a receptive audience for well-designed educational programs. Taken together, these findings illustrate how the public socially constructs ALS as a rare but serious and poorly understood condition, with high perceived severity but limited concrete knowledge (Caga et al., 2019; Cavallo et al., 2011). This pattern aligns with systems-thinking and problem-framing perspectives, in which public understanding, health system capacity, and policy responses interact to shape how a disease is recognized and prioritized within a community (Gadsby & Wilding, 2024; Organization WH, 2022). Viewing ALS awareness through this lens supports multi-level strategies that address not only individual knowledge gaps but also the broader informational and structural context depicted in our proposed action plan (Benatar et al., 2023; Howard et al., 2024; Lyndgaard, Storey & Kanfer, 2024).

Integrating ALS messages into broader neurological and rare disease awareness strategies, through schools, universities, primary care, and community media, could plausibly support timelier recognition, referral, and societal support, by analogy with similar initiatives in stroke and epilepsy, although our cross-sectional data cannot directly demonstrate such effects (Fig. 5) (Anonymous, 2025; Alzayer et al., 2023; Aytaç et al., 2014).

**Figure 5:**
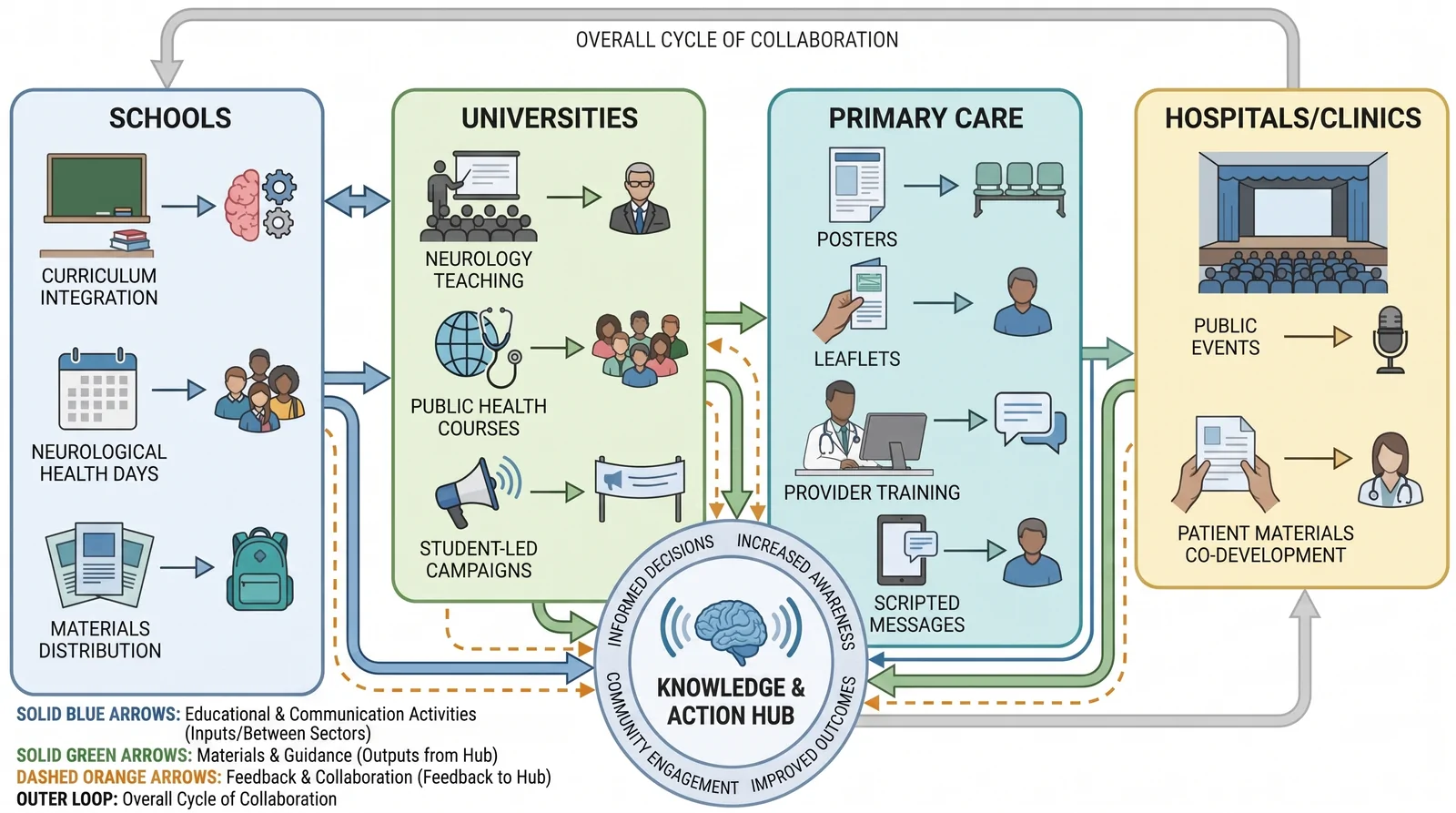
Proposed multi-level action plan to enhance ALS awareness. The diagram illustrates how schools, universities, primary care, and hospitals/clinics can each contribute educational and communication activities to a central “knowledge and action hub”, and receive materials and feedback in return. Arrows indicate the bidirectional flow of information and collaboration between sectors (for example, universities providing training content to primary care and the hub, and primary care feeding back community needs), rather than causal effects. Integrated implementation of these strategies is intended to support improved ALS knowledge and, in combination with accessible referral pathways, timelier recognition and referral of suspected cases. Generated by AI tool GAAbstract (https://gaabstract.com/) based on the authors’ edited text, and subsequently reviewed and modified by the authors to ensure scientific accuracy.

This study also adds a Saudi perspective to the global literature on ALS and MND awareness, which Western data have dominated (Liang et al., 2025; Park, Kim & Song, 2022). While previous Saudi work has focused primarily on provider-level barriers and inconsistent access to multidisciplinary ALS care, our findings demonstrate that upstream community awareness is also poor (Abuzinadah et al., 2020; Hogden et al., 2017). They may further hinder early diagnosis and advocacy. Together, these data add to the rationale for a coordinated national strategy that addresses both service provision and public education, recognising that improvements in public awareness must be accompanied by accessible pathways to neurological evaluation and multidisciplinary ALS care if earlier diagnosis and advocacy are to be achieved (Song, 2014).

Several limitations should be acknowledged when interpreting these results. The use of convenience sampling and an online component may limit generalizability, potentially over-representing younger and more educated individuals; if anything, this would bias awareness estimates upward, suggesting that the true community burden of low ALS literacy may be even greater. Moreover, although participants were recruited from public places and *via* a survey link distributed *via* social media platforms, the recruitment mode was not recorded at the individual level, so mode-specific differences in responses could not be examined. The cross-sectional design precludes causal inference, and self-reported awareness is subject to recall and social desirability biases. In addition, the multivariable model was developed primarily to explore associations rather than to construct a formal prediction tool; variable selection relied partly on *p*-value thresholds, which may lead to unstable estimates and potential overfitting. Nonetheless, the large sample size, the questionnaire’s robust psychometric structure, and the internally consistent performance of the exploratory multivariable results support the internal validity and practical relevance of the findings, while recognizing that variable selection based partly on *p*-values may introduce some instability and potential overfitting.

## Conclusions

In summary, this study demonstrates substantial gaps in ALS awareness and knowledge among adults in the Northern Border Region of Saudi Arabia. It confirms our hypothesis that overall awareness is generally poor. Prior exposure to ALS and the use of digital and healthcare information sources emerged as key correlates of greater awareness, whereas sociodemographic factors alone were weakly associated. These findings contribute to the regional data in the limited global literature on community awareness of ALS, but should be interpreted in light of the non-probability sampling and cross-sectional design, which may limit generalizability and preclude causal inference. Future research should evaluate targeted, digitally enabled, and clinician-led educational interventions and, where possible, assess whether improved ALS literacy is associated with earlier recognition, timelier referral, and better patient and caregiver outcomes, alongside efforts to strengthen healthcare pathways for rapid access to specialized neurological assessment.

## Supplemental Information

10.7717/peerj.21710/supp-1 Supplemental Information 1 Supplementary Data

10.7717/peerj.21710/supp-2 Supplemental Information 2 Questionnaire in English

10.7717/peerj.21710/supp-3 Supplemental Information 3 Supplementary Figures

## Additional Information and Declarations

### Data Availability

The following information was supplied regarding data availability: The raw data is available in the Supplemental Files.
