# Supplementary material for: Community awareness and knowledge of amyotrophic lateral sclerosis: a cross-sectional study in the Northern Border Region of Saudi Arabia
Source: PeerJ. 2026 Nov 5;14:e21710. doi: 10.7717/peerj.21710 (PMC13744703; doi:10.7717/peerj.21710)
Supplement: Supplementary file 1 [file peerj-14-21710-s002.pdf]

Community Awareness and Knowledge of Amyotrophic Lateral Sclerosis: A Cross-Sectional Study in the Northern Border Region of Saudi Arabia

Section 1 **Demographic Information**	**Question**	**Answer Options**
	1. Age	18-30<br>30-45<br>45-60<br>>60
	2. Gender	Male<br>Female
	3. Education level	Less than high school<br>- High school graduate<br>- College/university degree<br>- Postgraduate degree
	4. Occupation	Health sciences student<br>Non-health sciences student<br>Work (in the health field)<br>Work (out health field)<br>Not work
	5. Region of residence	…………………..

Section 2 **General Awareness**	**Question**	**Answer Options**
	1-Have you ever heard of Amyotrophic Lateral Sclerosis (ALS)?	• Yes<br>• No
	2-Is amyotrophic lateral sclerosis the same as multiple sclerosis	• Yes<br>• No<br>• I don't know
	3-Is amyotrophic lateral sclerosis the same as Parkinson's disease	• Yes<br>• No<br>• I don't know
	4-Have you heard of or taken part in the ALS Ice Bucket Challenge?	• Yes<br>• No
	5-How long is a person with ALS likely to live?	• 5 years<br>• 5-10 years<br>• Normal life expectancy<br>• I don't know
	6-Which gender is most affected by ALS?	• Male<br>• Female

		• I don’t know
	7-Which age group has the highest risk of developing ALS?	• <40<br>• 40-70<br>• >70<br>• I don’t know
	8-What is the etiology of ALS?<br>(Select all the options you expect)	• Genetic<br>• Lifestyle factors, e.g., obesity, smoking, drugs<br>• Infectious<br>• It is not known<br>• I don’t know
	9-ALS primarily affects which of the following?	• Muscles<br>• Brain and spinal cord<br>• Heart<br>• Skin
	10-Does ALS cause muscle weakness	• Yes<br>• No<br>• I don’t know
	11-Does ALS cause Dysarthria and Dysphagia	• Yes<br>• No<br>• I don’t know
	12-Does ALS cause difficulty breathing	• Yes<br>• No<br>• I don’t know
	13-Does ALS cause Loss of sensation	• Yes<br>• No<br>• I don’t know
	14-Does ALS cause memory loss	• Yes<br>• No<br>• I don’t know
	15-Is there a known cure for ALS?	• Yes<br>• No<br>• I don’t know
	16-Is the diagnosis of ALS difficult	• Yes<br>• No<br>• I don’t know

Section 3 **Attitudes and Perceptions**		
	How serious do you consider ALS?	- Not serious<br>- Slightly serious<br>- Moderately serious<br>- Very serious
	Do you think society provides enough support and resources for ALS patients	Yes<br>No<br>Not sure
	Do you consider there is stigma or social embarrassment associated with ALS in Saudi culture	Yes<br>No<br>Not sure
	Do you think there is enough information about ALS available through official health campaigns/media?	Yes<br>No<br>Not sure
	Would you be interested in learning more about ALS?	Yes<br>No<br>Not sure

Section 4 **Experience with ALS**		
	Do you know any famous person diagnosed with ALS in KSA?	-Yes<br>- No
	If yes, please mention their name.	……………
	Do you personally know anyone diagnosed with ALS?	- Yes<br>- No
	Have you ever cared for or supported someone with ALS?	- Yes<br>- No

Section 5 **Source of information**		
	Where did you hear about ALS?	-Internet<br>- TV/Radio<br>- Healthcare professionals<br>- Family or friends<br>- Social media<br>- Other (please specify):<br>……………_______________
