# Supplementary material for: Community awareness and knowledge of amyotrophic lateral sclerosis: a cross-sectional study in the Northern Border Region of Saudi Arabia
Source: PeerJ. 2026 Nov 5;14:e21710. doi: 10.7717/peerj.21710 (PMC13744703; doi:10.7717/peerj.21710)
Supplement: Supplementary file 2 [file peerj-14-21710-s003.pdf]

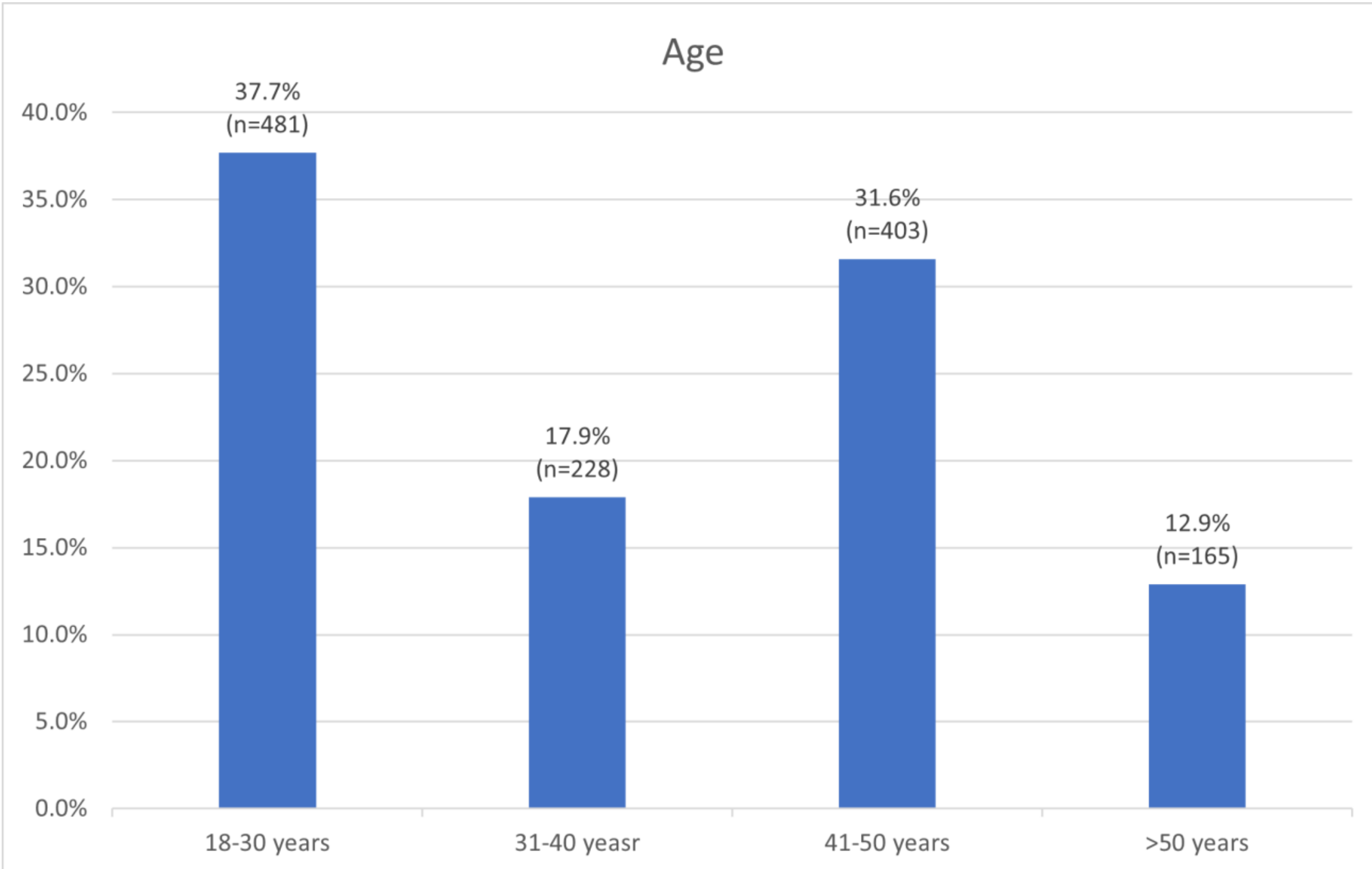

Fig S1. Age distribution of the study participants.

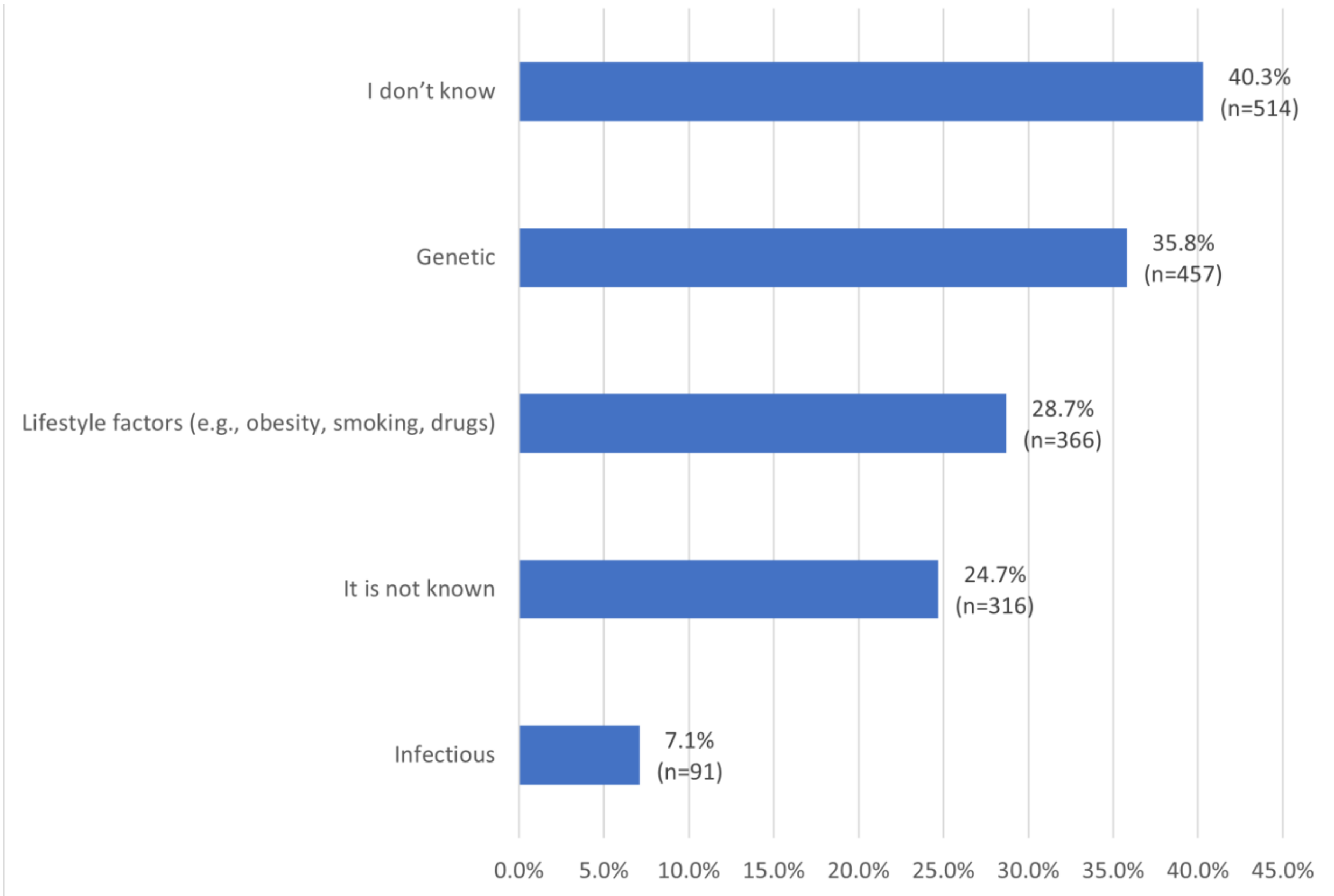

Fig S2. Perceived causes of als among participants

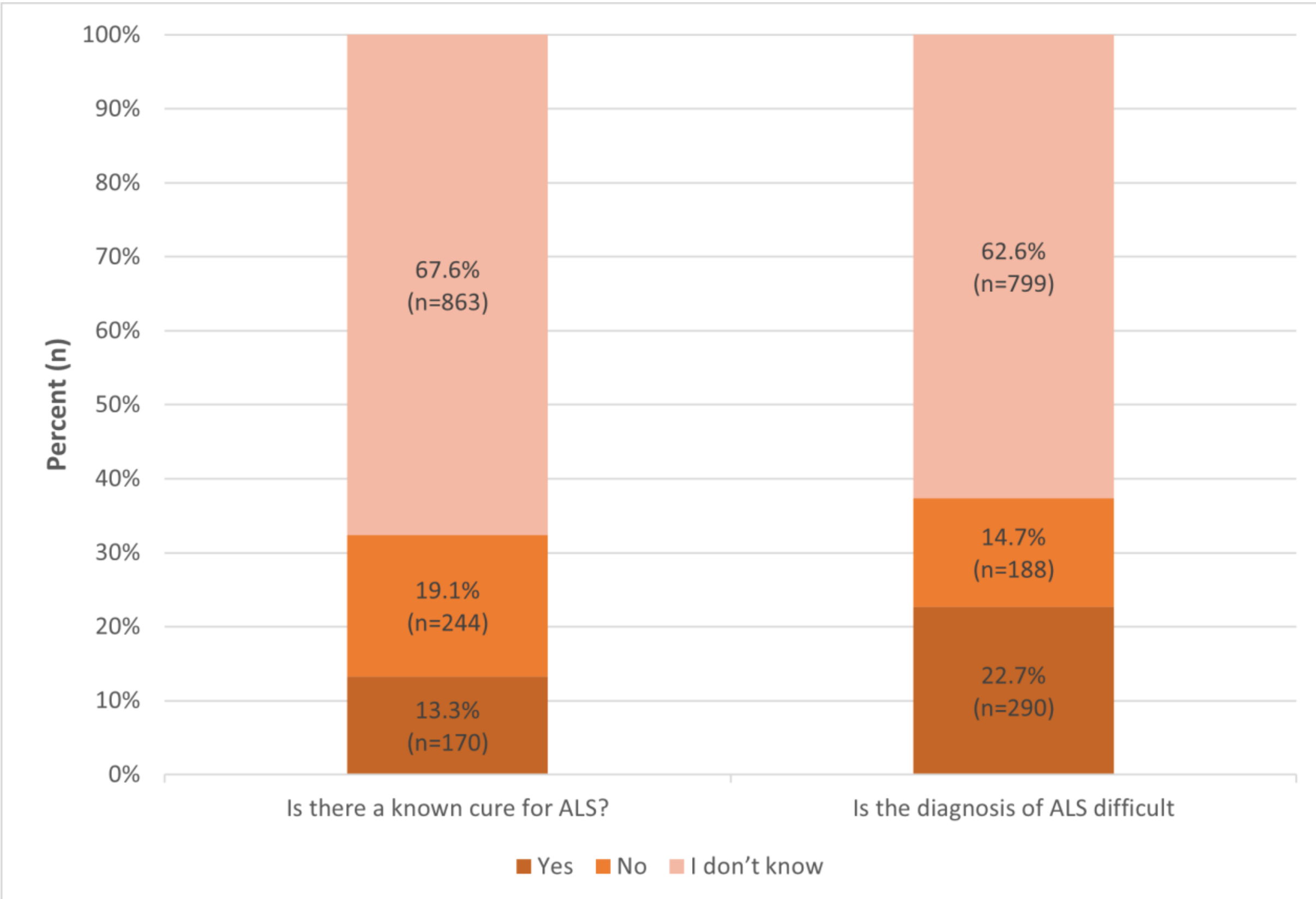

Fig S3. Participants' knowledge of ALS cure and the difficulty of diagnosis.

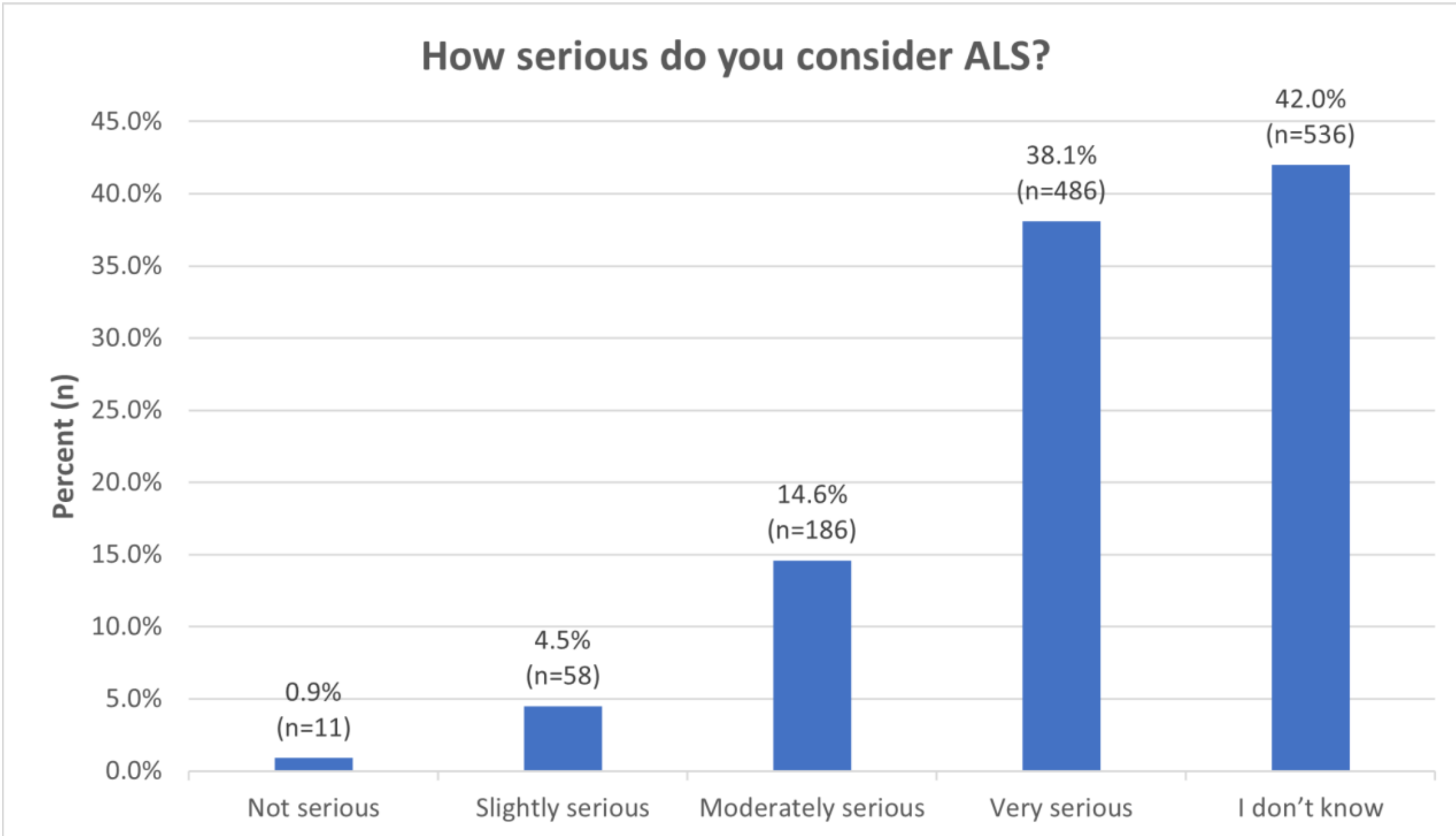

Fig S4. Perceived seriousness of ALS among participants.

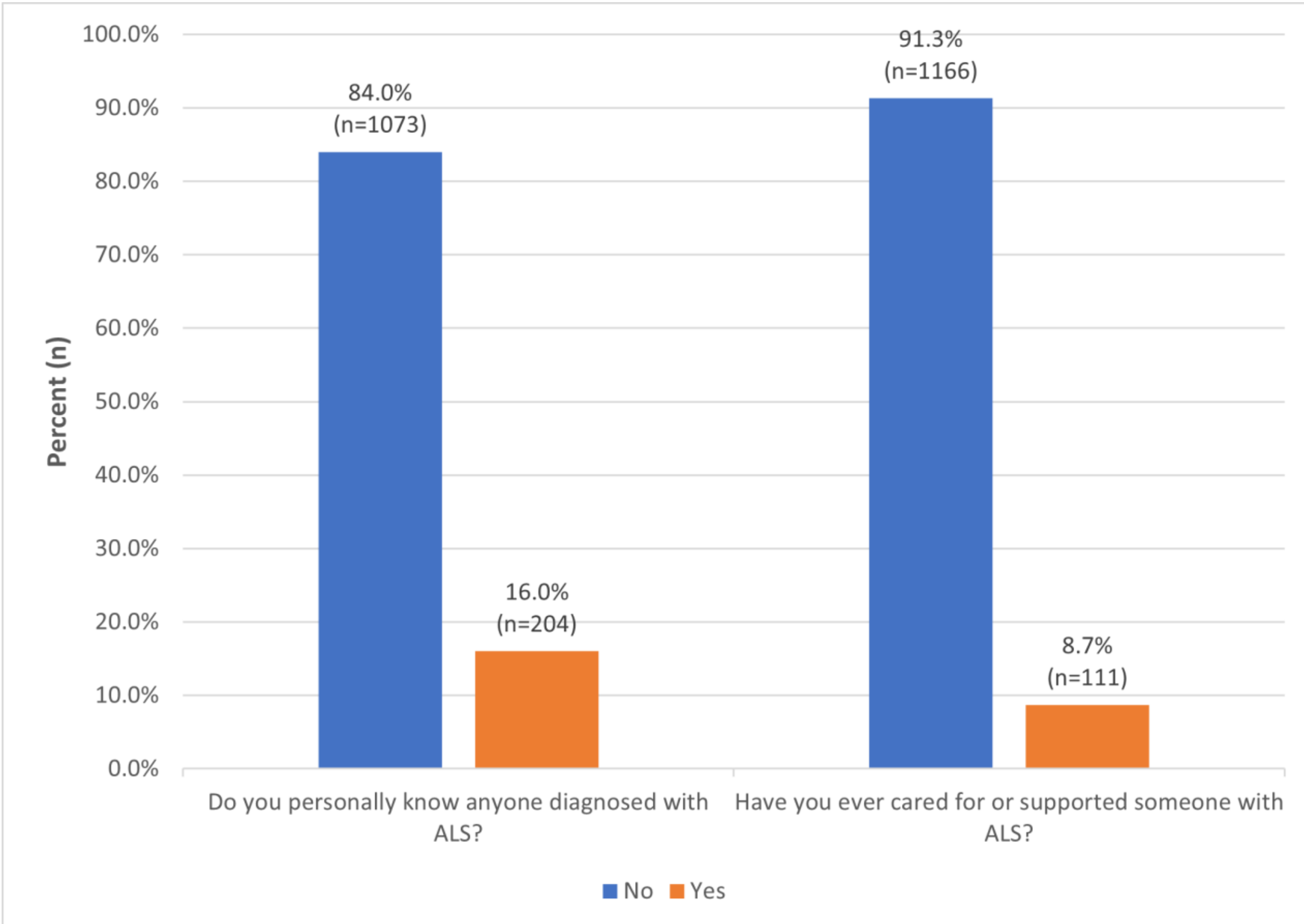

Fig S5. Personal experience with ALS among participants.

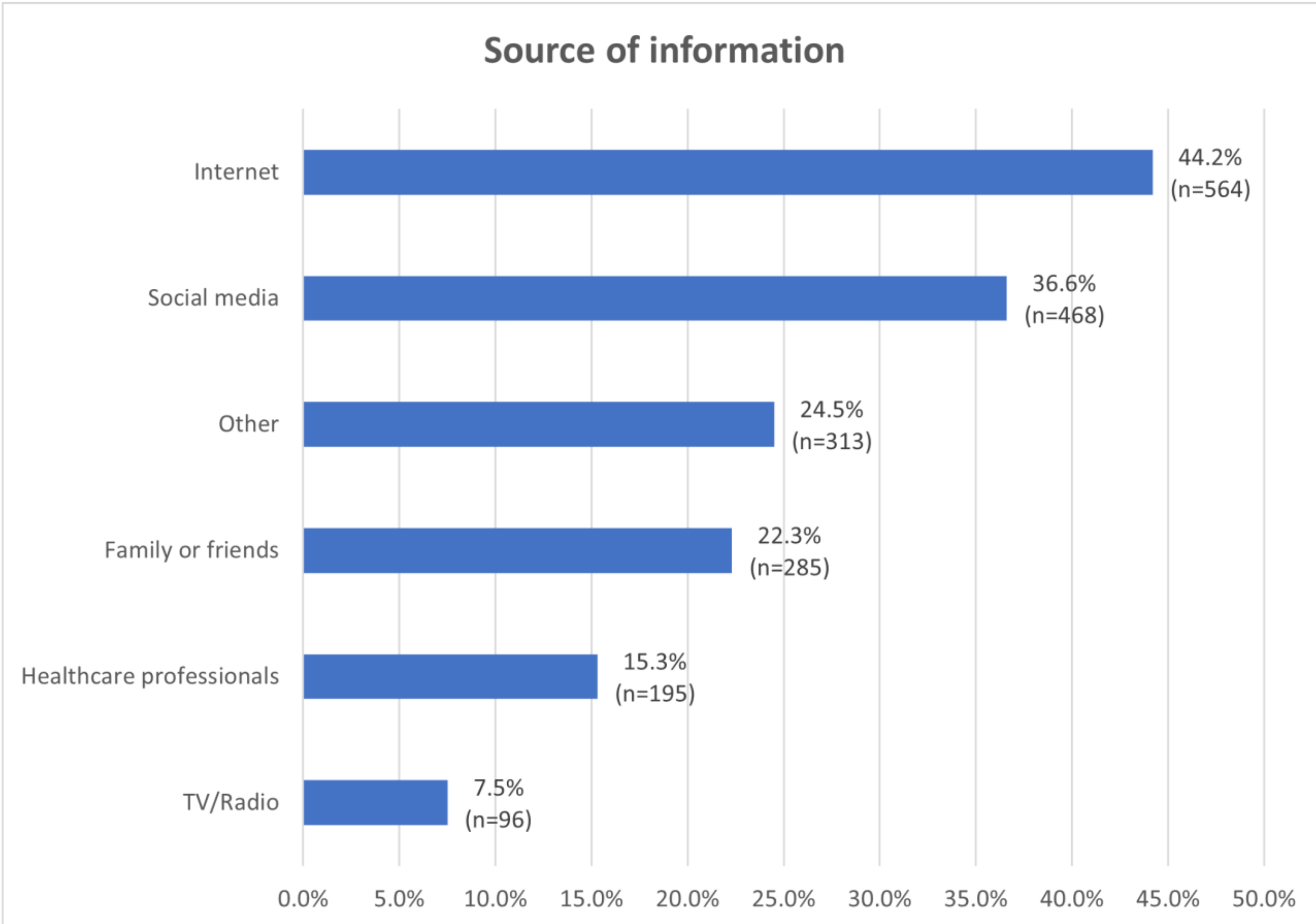

Fig S6. Sources of information about ALS reported by participants.
